# Dissipation behavior of octachlorodipropyl ether residues during tea planting and brewing process

**DOI:** 10.1007/s10661-016-5573-z

**Published:** 2016-09-07

**Authors:** Min Liao, Yanhong Shi, Haiqun Cao, Rimao Hua, Feng Tang, Xiangwei Wu, Jun Tang

**Affiliations:** 1 School of Plant Protection, Anhui Agricultural University, Hefei, 230036 China; 2 Provincial Key Laboratory for Agri-Food Safety, Hefei, 230036 Anhui China; 3 School of Resource & Environment, Anhui Agricultural University, Hefei, 230036 China

**Keywords:** Octachlorodipropyl ether (OCDPE), Residues, Dissipation, Fresh tea shoots, Prepared tea

## Abstract

The dissipation behavior of octachlorodipropyl ether (OCDPE) residues in fresh tea shoots and in tea prepared under field conditions was investigated, and the transfer of residues from brewed tea to tea infusion was determined. OCDPE levels in tea shoots, prepared tea, tea infusion, and spent tea leaves were determined using a sensitive and simple method. The dissipation of OCDPE is fairly slow in tea shoots and prepared tea, with half-life values of 5.10 and 5.46 days, respectively. The degradation rates of OCDPE residues in tea processing were 23.9–43.1 %. The terminal residues of OCDPE in tea shoots and prepared tea samples after 20 and 30 days of OCDPE application were higher than 0.01 mg/kg. However, OCDPE’s transfer rates from brewed tea to tea infusion were only 6.0–14.8 %. Further studies on risk assessment of OCDPE residue in tea on the basis of the relationship of OCDPE in prepared tea and infusion are warranted.

## Introduction

One of the most important non-alcoholic drinks, tea is the second most consumed beverage after water worldwide (Peng et al. 2016). Chinese were the first to use tea as medicinal drink, later as a beverage, and have been doing so for the past 3000 years (Zheng et al. 2016). At the present, China is ranked highest in tea planting and production and second for global tea exports (Hou et al. 2013a, b). Insect pests play an important role in the factors that limit the quality and quantity of tea production. It is imperative that such synthetic chemical pesticides as carbamates, organophosphates, and synthetic pyrethroids are applied to combat pests to attain high tea yields and economic returns. Therefore, it is of importance to understand pesticides’ behavior on tea bushes. Moreover, humans absorb the chemical compositions and residues through tea infusion when they drink tea as beverage (Karak and Bhagat 2010). Many papers focused on dissipation behavior of pesticides in the tea field, through manufacturing processes and through tea infusion (Hou et al. 2013a, b; Chen et al. 2013; Seenivasan and Muraleedharan 2009; Sharma et al. 2008).

**Fig. 1:**
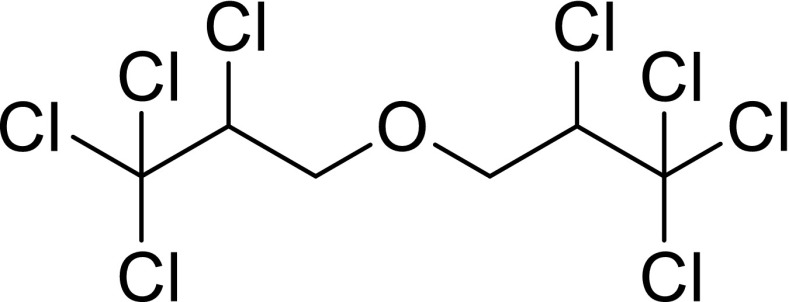
Structure of octachlorodipropyl ether (OCDPE)

Octachlorodipropyl ether [bis-(2,3,3,3-tetrachloropropyl) ether, OCDPE, CAS Registry No. 127–90-2, commercial name: S-2, S-421], is a chloroalkyl ether (Fig. 1) first prepared in 1959 (Friedrich et al. 1960) and soon discovered to have synergistic activity (Hayashi 1969). It is widely used in commercial agricultural and household insecticides as a result of its role as an insecticide synergist for pyrethroid, organophosphorus, and carbamate insecticides (Y. H. Shi et al. 2012). OCDPE has subacute or chronic toxicities such as subacute hepatotoxicity, cytotoxicity, carcinogenicity, and contact allergenicity, although its acute toxicity is low (Yoshida et al. 2001). Since the 1980s, it has been detected that OCDPE residues exist in human breast milk (Miyazaki et al. 1982), household dust (Yoshida et al. 1997), surface water, sediments, rain (Qing-Zhen 1996), and fish (Yoshida et al. 2001). More attention is suggested to be paid to OCDPE’s environmental behavior and other persistent organochlorine pesticides as well, such as HCHs, DDTs, and chlordane, which have long half-lives and therefore persist in the environment (Zhang et al. 2015).

Since the maximum residue limits (MRL) of OCDPE in tea were established by the EU in 2003 (Cai et al. 2003), OCDPE residues in tea have received extensive attention and was detected in many tea samples with high detection rates (Ding et al. 2007; MA et al. 2008; Zhao et al. 2008). Because OCDPE was widely used in household insecticides as a synergist, it was suggested that the main source of OCDPE in tea was the absorption from combustion of mosquito coils and the use of aerosols (Fubin et al. 2007; Huang et al. 2010). However, we analyzed 35 types of commercial insecticide formulations in the pesticide markets near the tea plantations and found that 72.7 % of the tested pyrethroid formulations contain OCDPE (Cao et al. 2007). Furthermore, there were significant residues of OCDPE detected in fresh tea shoots at tea plantations (Tang et al. 2007). Therefore, we suggested that the main source of OCDPE residue in tea was the usage of the insecticide formulations containing OCDPE during tea planting. The present work was carried out to develop sensitive analytical methods for OCDPE in different tea matrices (fresh tea shoots, prepared tea, tea infusion, and spent tea leaves) and to study the dissipation behavior of OCDPE residues during the process of tea planting and brewing, evaluate OCDPE’s persistence in tea, and the transfer from made tea to tea infusion and retention in spent tea leaves. This knowledge is useful for health risk assessments of OCDPE residues in tea and ascertaining OCDPE’s safety in tea plantations.

## Materials and methods

### Materials

A certified OCDPE standard (98.5 % purity) was obtained from Dr. Ehrenstorfer GmbH (Augsburg, Germany); technical grade OCDPE (93.0 % purity) was obtained from Zhejiang Changxing Zhongshan Chemical Industry Co., Ltd. (Zhejiang, China); emulsifier 2201 was obtained from Anhui Jintai pesticide chemical Co., Ltd. (Hefei, China); all of the reagents were analysis–grade and obtained from the Shanghai Chemical Reagent Corp., China Medicine Group (Shanghai, China). Aluminum oxide was activated at 550 °C for 4 h and deactivated with 5 % water prior to use. Sodium chloride (NaCl) and anhydrous sodium sulfate were dried at 550 °C for 4 h and stored in desiccators and dehydrated in an oven at 140 °C for 2 h before use.

The test of 10 % OCDPE EC (emulsifiable concentrate) was laboratory-prepared by mixing 5.40 g of technical grade OCDPE with 5.0 g emulsifier 2201 and 46.4 g solvent (Cao et al. 2007), which was fit for the EC formulation standard.

Stock solutions of 100 μg ml^−1^ of OCDPE were prepared in hexane. Working standard solutions were prepared by diluting the stock solution with hexane. All solutions were preserved at 4 °C.

### Field trials and sampling

The field trials, consisting of a dissipation experiment and a final residue experiment, were conducted in 2010 at the tea plantation of Tianhu town in Ningguo city, Anhui Province, China. Based on the guidelines on pesticide residue trials, each experimental field was composed of three replicate plots with an area of 35 m^2^ for the control and for the different OCDPE treatment. Two untreated guard rows partitioned and isolated the plots from one another.

The dissipation experiment was performed at a higher dosage level of 10 % OCDPE EC (75 g a.i. ha^−1^), double the proposed recommended dosage, which was estimated by the recommended dosage of pyrethroids used in tea, on average; the amount of OCDPE is two to ten times the amount of active ingredient in pyrethroid formulations (Chao et al. 2006), and three untreated pots were sprayed with water as control. Approximately 2.0 kg of fresh tea shoots with a bud and two leaves was picked from each treated and control plot, and brought to the laboratory at each time point: 0 (0.5 h after spraying) 1, 3, 5, 7, 10, 15, 20, 25, 30, and 35 days after treatment.

The final residue field test was designed similar to the above test, but OCDPE was sprayed at two doses, i.e., a normal dosage of 37.5 g a.i. ha^−1^(the proposed recommended dosage) and a high dosage of 75 g a.i. ha^−1^ (double the proposed recommended dosage), respectively. Each dosage application was sprayed two and three times. Three identical procedures were carried out at each treatment and each plot was separated by a buffer area. The spraying interval was 7 days. Approximately 2.0 kg of the fresh tea shoots with a bud and two leaves were picked from each treated and controlled plot and brought to the laboratory at 20 and 30 days after spraying.

### Tea leaf processing and infusion preparation

Fresh tea shoot samples from the dissipation field trial were processed into green tea using the standard steps (spreading, fixing, rolling, and baking). The fresh tea shoots and prepared tea samples at different intervals were collected to determine the presence of OCDPE residue at the same time.

The prepared tea samples were taken at different intervals during the dissipation field trial with different concentrations of OCDPE and were subjected to the infusion experiments. Thirty grams of prepared tea of each sample was divided into six equal parts, three of which were used to determine the OCDPE residue (three replications), and the other three parts were subjected to the infusion process. Briefly, prepared tea (5.0 g) was infused in 150 mL of boiled water. After 30 min of brewing, the water extract was filtered, and the spent leaves were infused in another 150 mL of boiled water. The filtrate was collected in the same beaker, cooled, and examined for OCDPE residue transfer. The spent leaves were also collected for the determination of OCDPE residue.

### Analytical methods

#### Extraction and clean-up procedure from fresh tea shoots and spent leaves

Fresh tea shoots (20 g) and spent leaves (20 g) were extracted with 80 ml acetone + hexane (2:1 *v*/*v*) by mechanical shaking for 1 h. Extracts were filtered through a Buchner funnel and transferred into a 500 mL separatory funnel, to which 20 mL of hexane and 100 mL of 5 % aqueous NaCl were added. After perfect blending, the aqueous layer was separated and the organic layer was transferred into a 150 mL round bottom flask. The aqueous phase was partitioned with hexane (20 ml × 2). The extract was collected in the same round bottom flask, evaporated at 40~45 °C to near 2 mL, and then transferred to an aluminum oxide column (20 cm × 1.0 cm i.d.; 5 g of aluminum oxide (60–100 mesh) thoroughly mixed with 0.1 g activated carbon) and prewashed with 30 mL acetone/hexane (1/1, *v*/*v*). The column was eluted with 50 mL of acetone + hexane (1:4 *v*/*v*). The eluent was concentrated to dryness. The residue was reconstituted in 5 mL of hexane for GC-ECD analysis.

#### Extraction and clean-up procedure from prepared tea

Prepared tea (5.0 g) was extracted with 30 ml acetone + hexane (2:1 *v*/*v*) by mechanical shaking for 1 h. Extracts were filtered through a glass funnel containing 5 g of anhydrous sodium sulfate into a 150 mL round bottom flask, evaporated under vacuum to dryness at a bath temperature of 40 °C, and then cleaned with an aluminum oxide column as described above.

#### Extraction from tea infusion

A 100-mL aliquot of the cooled infusion extract was partitioned into 20 mL of hexane three times. The organic layer was then separated and filtered through a glass funnel containing 5 g of anhydrous sodium sulfate into a 150 ml round bottom flask, evaporated under vacuum to dryness at a bath temperature of 40 °C, and then cleaned with an aluminum oxide column as described above.

#### GC determination

The concentration of OCDPE was obtained by gas chromatography (GC) using an Agilent 6890 instrument equipped with a ^63^Ni electron capture detector (ECD) while the OCDPE was identified using an Agilent Technologies 5977A mass spectrometer operating in the EI mode at 70 Ev, equipped with a splitless injector (250 °C). The GC settings were the same as those used our previously reported method (Y.-h. Shi et al. 2015).

## Results and discussion

### Method validation

The described method used to determine OCDPE residues in tea shoots, prepared tea, tea infusions, and spent leaves by GC-ECD is sensitive and selective. Quantification was accomplished using a standard curve, prepared by diluting the stock solution in hexane. Good linearity was achieved with a correlation coefficient of 0.9935. The limit of quantification (LOQ) was determined as 0.01 mg kg^−1^ for tea shoots, prepared tea, and spent leaves, and 0.1 μg L^−1^ for tea infusions when the signal to noise ratio was 10:1. No control samples showed any evidence of chromatographic interference.

Recovery and relative standard deviation (RSD) were used to estimate accuracy and precision at three levels, namely, 0.01, 0.05, and 0.5 mg kg^−1^ for tea shoots, prepared tea, and spent leaves, and 0.1, 0.5, and 5.0 μg L^−1^ were used for tea infusions (five times each). Table 1 shows the results of the recovery study of OCDPE in tea shoots, prepared tea, tea infusions, and spent leaves, which were 82.3–97.7, 89.4–105.6, 78.2–98.3, and 83.5–92.1 % with relevant RSDs of 8.0–10.2, 5.2–8.2, 6.9–9.3, and 6.3–9.2 %, respectively.

**Table 1:** Recoveries and relative standard deviations (RSDs) of OCDPE in the fortified samples

Sample	Fortified level μg/kg^a^	Average recoveries^b^ (%)	Standard deviation	RSD (%)
Tea shoots	10	82.3	8.4	10.2
	50	84.3	6.8	8.0
	500	97.7	9.1	9.4
Prepared tea	10	105.6	8.7	8.2
	50	89.4	6.8	7.6
	500	90.4	4.7	5.2
Tea infusions	0.1	98.3	9.1	9.3
	1.0	85.7	5.9	6.9
	5.0	78.2	6.9	8.8
Spent leaves	10	88.6	7.2	8.1
	50	92.1	5.8	6.3
	500	83.5	7.7	9.2

^a^The unit of OCDPE in tea infusions is μg L^−1^

^b^Five replicate extractions were performed for each treatment

### Dissipation of OCDE residues in fresh tea shoots and made tea

**Fig. 2:**
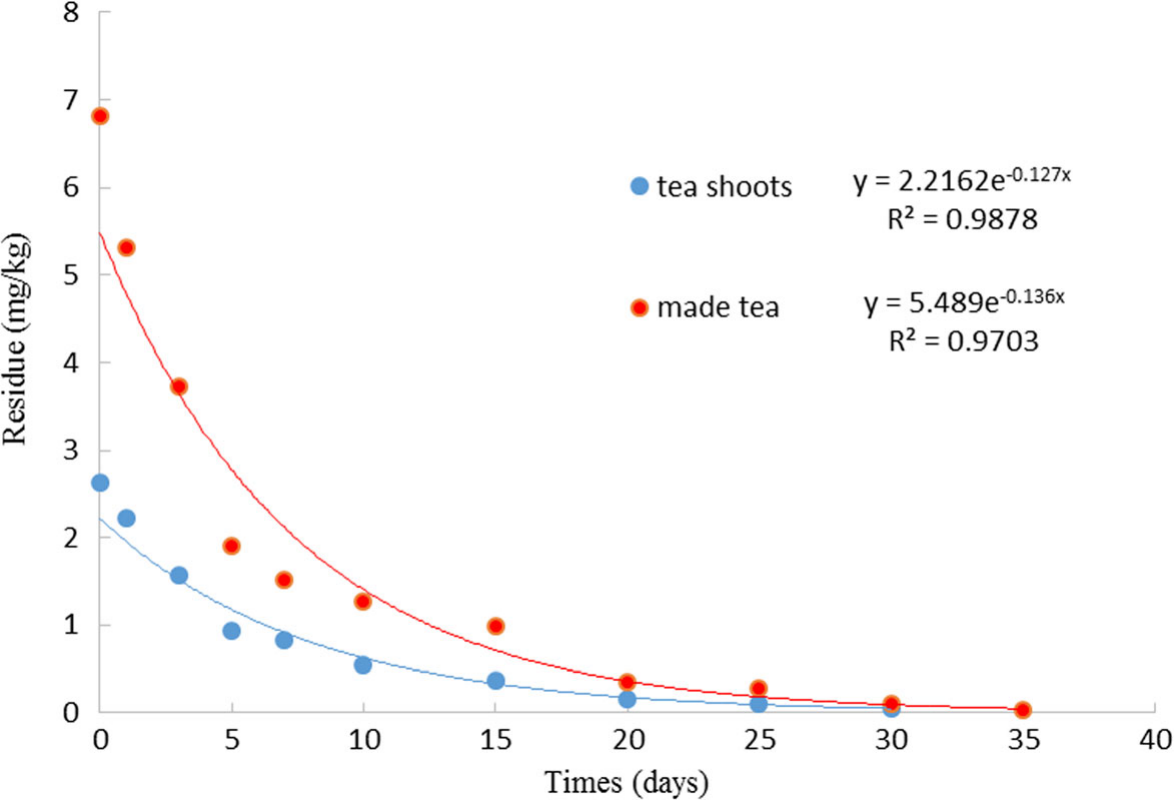
The dissipation curve of OCDPE in tea shoots and prepared tea

The results of the OCDPE residue analysis and the percent dissipation in tea shoots at different intervals are presented in Table 2. No residues of OCDPE were detected in any analyzed control tea sample. The initial mean deposit of OCDPE in tea shoots at the dosage of 75 g a.i. ha^−1^ was 2.63 mg kg^−1^. The residues dropped to 0.55 mg kg^−1^ in the 10th day sample, dissipated by >90 % after 20 days, and could not be detected in the 35th day sample under the treatments. The OCDPE residues in prepared tea were 1.8–2.7 times higher than the levels in the corresponding samples of fresh tea shoots for the OCDPE. The dissipation trend of OCDPE in tea shoots and made tea followed first order kinetics. The persistence patterns are presented in Fig. 2. The half-life values of OCDPE were 5.46 and 5.10 days in tea shoots and prepared tea, indicating that OCDPE dissipated more slowly after application because the half-life values of many pesticides dissipated in tea crops ranged from 1.58 to 3.85 days (Paramasivam and Chandrasekaran 2014; Kashyap et al. 2015; Kaur et al. 2015).

**Table 2:** OCDPE residues in tea shoots and prepared tea at different time intervals (*n* = 3)

Internal days (d)	Tea shoots		Made tea		Degradation in tea processing (%)^a^
	OCDPE residue (mg/kg ± SD)	Moisture content (% ± SD)	OCDPE residue (mg/kg ± SD)	Moisture content (% ± SD)	
0	2.63 ± 0.25	72.2 ± 1.08^b^	6.82 ± 0.71	5.27 ± 0.15	23.9
1	2.23 ± 0.25 (15.2)^c^	73.1 ± 1.32	5.32 ± 0.52 (22.0)	5.29 ± 0.12	32.2
3	1.57 ± 0.15 (40.3)	72.5 ± 1.01	3.72 ± 0.32 (45.5)	4.97 ± 0.11	31.4
5	0.93 ± 0.12 (64.6)	71.8 ± 1.13	1.91 ± 0.21 (72.0)	4.93 ± 0.12	39.1
7	0.82 ± 0.11 (68.8)	70.6 ± 1.21	1.51 ± 0.16 (77.9)	4.87 ± 0.09	43.1
10	0.55 ± 0.06 (79.1)	71.9 ± 1.35	1.27 ± 0.11 (81.4)	4.69 ± 0.07	31.9
15	0.37 ± 0.03 (85.9)	73.9 ± 1.51	0.99 ± 0.09 (85.5)	4.98 ± 0.11	26.5
20	0.15 ± 0.01 (94.3)	74.1 ± 1.27	0.35 ± 0.03 (94.9)	5.11 ± 0.12	36.3
25	0.11 ± 0.01 (95.8)	74.9 ± 1.32	0.28 ± 0.02 (95.9)	5.08 ± 0.08	32.7
30	0.05 ± 0.00 (98.1)	73.3 ± 1.09	0.11 ± 0.01 (98.4)	5.13 ± 0.10	38.1
35	ND	73.5 ± 1.08	0.03 ± 0.00 (99.6)	5.02 ± 0.12	–

*ND* not detected

^a^Percentage of degradation in green tea processing was calculated as the decay ratio of OCDPE residue in dry matter of tea shoots and prepared tea

^b^Moisture content of tea shoots and prepared tea was conducted at “103 ± 2 °C constant weight method.” Three replications for each sample

^c^Percentage degradation after spraying

The following are factors affecting pesticide’s persistence in crops: the physical and chemical properties of the pesticide (chemical stability, water solubility, and volatility), the environmental factors (light, temperature, pH, and moisture) and the crop characteristics, such as the growth dilution factor (Liu et al. 2014; Xavier et al. 2014; Jacobsen et al. 2015). The physical and chemical properties of the pesticide might have played a basic role in the degradation of the insecticides. From above, it is proposed that OCDPE is fairly persistent in the crop and the environment, and this warrants further study.

Tea leaves were dehydrated during processing, causing a concentration factor of three to four fold (Gupta and Shanker 2009; Lavelli and Scarafoni 2012). In the present study, the moisture content of each of the fresh tea shoots and prepared tea samples was determined, and the dissipation rates in green tea processing were calculated with the OCDPE residue in the dry matter of prepared tea and tea shoots. As shown in Table 2, the degradation rates of OCDPE residues in tea processing were 23.9–43.1 %; the mean value of which was 33.5 %.

Evaporation and thermo-decomposition are the two main mechanisms of pesticide degradation during tea processing (Zongmao and Haibin 1988). The high vapor pressure and thermal decomposition may be the key factors for OCDPE degradation during tea processing.

### Terminal residues of OCDPE in fresh tea shoots and made tea

Table 3 summed up the terminal residues of OCDPE in fresh tea shoots and prepared tea samples collected from the treated plots. The results indicated that at a lower dosage of OCDPE, the terminal residues ranged from 0.018–0.172 mg/kg in fresh tea shoots and 0.085–0.517 mg/kg in prepared tea, respectively. At a high dosage, the terminal residues ranged from 0.063 to 0.313 mg/kg in tea shoots and 0.221–0.986 mg/kg in prepared tea. The collection time after application also affected the residue of OCDPE in tea shoots, which ranged from 0.118 to 0.313 and 0.018–0.097 mg/kg when the tea shoots sample was collected after 20 and 30 days of OCDPE application, respectively. In the prepared tea, the residue ranged from 0.276 to 0.986 and 0.085–0.378 mg/kg after 20 and 30 days of OCDPE application, respectively. As collection time increased, the residues obviously declined.

**Table 3:** The terminal residues of OCDPE (mg/kg) in tea shoots and prepared tea (*n* = 3)

Samples	Dose (g a.i. ha^−1^)	Number of times sprayed	Time after the last application	
			20 days	30 days
Fresh tea	37.5	2	0.118 ± 0.011	0.018 ± 0.002
		3	0.172 ± 0.019	0.039 ± 0.007
	75	2	0.271 ± 0.028	0.063 ± 0.010
		3	0.313 ± 0.039	0.097 ± 0.012
Prepared tea	37.5	2	0.276 ± 0.016	0.085 ± 0.008
		3	0.517 ± 0.051	0.133 ± 0.011
	75	2	0.603 ± 0.059	0.221 ± 0.017
		3	0.986 ± 0.063	0.378 ± 0.025

Regardless of the dose or the number of times sprayed, the residues of OCDPE in prepared tea after 20 and 30 days of OCDPE application were above 0.01 mg/kg, which is the MRL value established by the EU and Japan (China has not set an MRL value for OCDPE in tea). It showed that when OCDPE was used under the designed experiment, significant pesticide residue was detected in prepared tea, in amounts outside the acceptable limits of residual levels. It is worth mentioning that China has banned the sale of pesticides containing OCDPE, so it is unlikely that the OCDPE residues in the tea are due to the application of pesticide containing OCDPE. However, we found that there were significant residues of OCDPE in the soils of the tea plantations (six samples detected from 23 samples), and these residues could be transferred into the tea shoots (these results will be presented in another paper). It is thus evident that the residues of OCDPE in prepared tea and the soil of tea plantations, as well as its health risk assessment, should be areas of continuous focus.

### Leaching efficiency of OCDPE from prepared tea into tea infusion

Sixteen prepared tea samples at different intervals from the dissipation field trial were subjected to the infusion experiments. The OCDPE residues in prepared tea, the tea infusion, and the spent leaves were simultaneously determined and the data are presented in Table 4. Results reveal that the OCDPE residues in selected prepared tea samples ranged from 0.08 to 6.96 mg/kg. After tea brewing, OCDPE residues in the tea infusion and the spent leaves were 0.10–17.2 μg/L and 0.05–6.03 mg/kg, respectively. The transfer rates of OCDPE from prepared tea to the infusion were 6.0–14.8 %; the mean value of which was 8.98 %. The transfer efficiency of the pesticide residue into the infusion depends on its water solubility, partition coefficient, and low vapor pressure (Iizuka and Shimizu 2014; Fantke et al. 2012; Houbraken et al. 2016). Based on the present research, the lower transfer rate of OCDPE from prepared tea to the infusion may be due to its hydrophobic character (insolubility in water) and high vapor pressure.

**Table 4:** Transfer of OCDPE residue into the tea infusion (*n* = 3)

No.	Tea infusion		Spent leaves		Prepared tea		Transfer percent to infusion (%)
	Amount (μg)	Concentration (μg/L)	Amount (μg)	Concentration (mg/kg)	Amount (μg)	Concentration (mg/kg)	
1	5.16	17.2 ± 1.15	30.1	6.03 ± 0.52	34.8	6.96 ± 0.36	14.8
2	4.32	14.4 ± 0.87	31.7	6.34 ± 0.44	33.1	6.61 ± 0.48	13.1
3	2.76	9.19 ± 0.56	19.9	3.98 ± 0.31	26.7	5.34 ± 0.33	10.3
4	2.20	7.34 ± 0.61	17.1	3.42 ± 0.28	17.8	3.56 ± 0.31	12.4
5	1.16	3.86 ± 0.19	8.80	1.76 ± 0.11	9.75	1.95 ± 0.15	11.9
6	0.75	2.50 ± 0.15	7.35	1.47 ± 0.09	7.80	1.56 ± 0.12	9.62
7	0.52	1.74 ± 0.11	4.90	0.98 ± 0.06	6.15	1.23 ± 0.11	8.49
8	0.39	1.30 ± 0.09	4.45	0.89 ± 0.06	4.90	0.98 ± 0.05	7.96
9	0.35	1.17 ± 0.07	4.80	0.96 ± 0.05	4.10	0.82 ± 0.06	8.56
10	0.13	0.44 ± 0.02	2.25	0.45 ± 0.03	2.20	0.44 ± 0.03	6.00
11	0.12	0.41 ± 0.02	1.75	0.35 ± 0.02	1.90	0.38 ± 0.02	6.47
12	0.10	0.32 ± 0.02	1.25	0.25 ± 0.02	1.45	0.29 ± 0.02	6.62
13	0.08	0.26 ± 0.01	1.05	0.21 ± 0.01	1.15	0.23 ± 0.01	6.78
14	0.07	0.23 ± 0.01	0.80	0.16 ± 0.01	0.95	0.19 ± 0.01	7.26
15	0.04	0.13 ± 0.01	0.55	0.11 ± 0.01	0.65	0.13 ± 0.01	6.00
16	0.03	0.10 ± 0.00	0.25	0.05 ± 0.00	0.40	0.08 ± 0.00	7.50

Exposure assessment is the most important step in risk assessment of pesticide residue in food (Wolejko et al. 2016). As tea is steeped prior to consumption, pesticide residues in prepared tea would be absorbed by humans through the tea infusion (Karak and Bhagat 2010). Therefore, the transfer rate of pesticide residue from prepared tea to the infusion is of great interest if we desire an estimate exposure assessment and the safety of pesticide residue in tea (Hou et al. 2013a, b; Chen et al. 2015). According to the theory of risk assessment, distribution probability models of tea consumption, body weight of tea drinkers, and OCDPE residue in prepared tea should be developed. Subsequently, on the basis of the relationship of the residues of OCDPE in the tea infusion and prepared tea, a distribution probability model of OCDPE exposure in prepared tea to tea drinkers could be developed by the Monte-Carlo method (Callahan 1996; van der Voet et al. 2009). In this study, the relationship of OCDEP residue in prepared tea (*X*, mg/kg) and the tea infusion (*Y*, μg/L) was best modeled by a quadratic function of the form *Y* = 0.1432*X* ^2^ + 1.3004*X* + 0.0055 (*R* ^2^ = 0.9851), when the residues of OCDPE in prepared tea were below 7.0 mg/kg. The risk assessment of OCDPE residue in tea for the health of tea drinkers is under study.

## Conclusion

OCDPE levels in fresh tea shoots, prepared tea, tea infusion, and spent leaves were determined with a sensitive and simple GC-ECD method. The dissipation and terminal residues in tea under field conditions were investigated, and the results showed that OCDPE dissipated fairly slowly in tea shoots and prepared tea; the half-life values of which were 5.10 and 5.46 days, respectively. The degradation rates of OCDPE residues during tea processing were 23.9–43.1 %; the mean value of which was 33.5 %. The terminal residues of OCDPE in fresh tea shoots and prepared tea samples after 20 and 30 days of OCDPE application were found to be higher than 0.01 mg/kg, which is the MRL value established by EU and Japan. However, the transfer rates of OCDPE from prepared tea to the infusion were 6.0–14.8 %, the mean value of which was 8.98 %. The relation of OCDPE residue in prepared tea (*X*, mg/kg) and the tea infusion (*Y*, μg/L) followed a quadratic function of *Y* = 0.1432*X* ^2^ + 1.3004*X* + 0.0055 (*R* ^2^ = 0.9851), when the residues of OCDPE in made tea were below 7.0 mg/kg. Further studies on the risk assessment of OCDPE residues in made tea for the health of tea drinkers are warranted.
